# Editorial: Engaging Women and Girls in Community Sport: Building an Equitable and Inclusive Future

**DOI:** 10.3389/fspor.2022.947626

**Published:** 2022-06-13

**Authors:** Meghan Casey, Alison Doherty, Sam K. Elliott, Leanne Norman

**Affiliations:** ^1^Institute of Health and Wellbeing, Federation University Australia, Ballarat, VIC, Australia; ^2^School of Kinesiology, Faculty of Health Sciences, Western University, London, ON, Canada; ^3^College of Education, Psychology and Social Work, SHAPE Research Centre, Flinders University, Adelaide, SA, Australia; ^4^Centre for Social Justice in Sport and Society, Carnegie School of Sport, Leeds Beckett University, Leeds, United Kingdom

**Keywords:** community sport and recreation, women and girls, equality, inclusion, participation, leadership

Women and girls are traditionally underrepresented in community sport and have faced a multi-level and complex set of barriers to their participation and leadership. Many women and girls still face structural, cultural and social barriers and depending on their intersectional identity they may also face oppression and marginalization (LaVoi, 2016). Positively, there are increasingly more opportunities for women and girls to play and lead within community sport; especially in traditionally male-dominated sports. However there are still prevailing attitudes that women/girls are “second rate” compared to men/boys and there is discriminatory allocation and access to resources (Adams and Leavitt, 2018; Casey et al., 2019; Eime et al., 2021). Uncovering the narratives surrounding the various issues from a range of disciplinary perspectives, country contexts and sports can inform building an equitable and inclusive future to attract and retain women and girls in community sports. Likewise, supporting community sport to develop ideas, methods and solutions with stakeholders informs practice and policy, like the development of a theoretical blueprint for attracting and retaining girls' in Australian football (Elliott et al., 2020) and netball clubs' utilizing fun and enjoyment to promote inclusion, participation and retention (Litchfield and Elliott, 2020).

In this Research Topic, seven teams of authors provide insights to women and girls' engagement as a platform for continuing to build a more equitable and inclusive future in community sport. The articles address women and girls' engagement as participants (see Eime et al.; Drummond et al.; Gjesdal and Hedenborg; Murray et al.; Rich et al.;) and women as coaches and officials (see Drury et al.; Hogan et al.). The collection showcases various issues continuing to face women and girls, as well as the policies, strategies and programs within community sport that are attempting to close the gender gap by overcoming persistent multi-level and complex structural, social and cultural barriers faced by women and girls in this particular context.

## Sport Participants

Eime et al. examined changes in participation trends across ten sports in Australia over a five-year period. They report that the gender gap between women/girls and men/boys might be gradually closing, and these changes are in line with sport policy strategies and investments focused on wider sport participation opportunities (e.g., male dominated sports), building female friendly infrastructure, and facilitating female sport leadership. Nuanced trends by gender and age are also presented. The authors note that the change in participation trends was only significant over the 5-year period, as opposed to annually, highlighting the time it takes to build a more equitable future. They put forth a call for more longitudinal studies of gender-based (and intersectional) participation trends, and systematic consideration of the impact of long-term sport policies and strategies on those trends.

Rich et al. used spatial mapping to analyze sport participation of women and girls in Ontario, Canada and the rural and urban nature of participant sports membership. They employed a novel spatial analysis technique to analyze broader participation trends and patterns, functional regions and participation data to categorize community context of women and girls' involvement in the sport of rowing, specifically. In reviewing membership data from Provincial Sport Organizations (PSO) with respect to gender, Rich et al. found that on average women represented 60% of PSO members in any given year (between 2014 and 2019 inclusive). The authors note that by understanding and mapping contextual patterns of participation, refined analysis of community sport contexts can enhance future sport development and policy initiatives designed to increase girls and women's sport participation.

Developing effective pathways into and across community sport in South Australia was the drive for the Drummond et al. examination of intrapersonal, interpersonal and environmental influences on community sporting pathways for girls and young women. They undertook a comprehensive research approach, applying a socio-ecological model to unpack barriers and facilitators, and identify solutions to attract and retain girls in sport. Mixed methods were used, surveying 2,189 high school students (12–18 years), along with focus group and interview data collected from a subset of 37 high-school students, parents and teachers. Male sport participants were included to juxtapose findings and obtain contrasting insights. The findings of this study showed it was important to intervene before school Year 10 to improve girls' involvement in sport (whilst Year 12 for boys). Importantly, the authors highlight the importance of focusing on what girls' bodies can do, rather than how they look to negate body image concerns and cultivate psychological resilience. The authors also note that fostering a delicate blend of social and development forces (e.g., friendships and sport skills) were key to keeping girls' in the sporting pathway. This contributes to the theoretical understanding of factors that support and inhibit girls' involvement in community sport.

Gjesdal and Hedenborg examined how a sport project succeeded at engaging minority girls, living in Norway, in organized community sport. They found value in combining the study of motivation using social determinants theory (SDT) with an ecological perspective to address social inequities, especially those connected to access to sport (financial support and transport). Utilizing a case study design, the authors were able to undertake an in-depth analysis of how the particular sport project succeeded through satisfying the basic psychological needs of the participants. These were principally a sense of inclusion within and facilitation through the sport environment. The authors concluded that to build girls' engagement, through a more equitable sporting environment, it is crucial to align participation within sporting programmes with girls' cultural norms and values, especially at the start of any initiative. It is also important to focus on other factors besides motivation to participate, such as the financial costs of being involved in such a programme. Finally, the success of community sporting programmes that aim to engage different groups of girls should also seek to include all girls, regardless of ability, to provide them with a sense of mastery and belonging.

Social factors are commonly reported to influence the motivation of girls to play sport, but knowledge of associations between the community sport team environment and individual processes have been lacking. Murray et al. partnered with Badminton Canada, hypothesizing that social identity and physical self-concept were important concepts related to team environment and would influence the motivation of girls to play mixed-gendered badminton. A total of 95 girls playing community badminton completed a self-report survey measuring social identity, physical self-perceptions, and motivation. The findings highlight the importance of team dynamics as girls who identify with their team reported better perceptions of their physical self, and this related to higher levels of autonomous motivation and lower levels of controlled motivation. The authors provide practical implications for team sports to consider team level strategies that develop a sense of “us” and belongingness among participants to keep girls playing sport. Further research is recommended to develop and test specific strategies that can improve the team environment to keep girls involved in community sport.

## Coaching and Officiating

In England, Drury et al. explored the experiences of women involved in non-playing roles (coaching and refereeing) in the male-dominated sport of football. They highlight the insidious and persistent nature of gendered microagression, the sexism of football culture, and the ways in which these women negotiate this masculine terrain in their pursuit of being coaches and referees. Drury et al. developed three substantive and contexualised themes to evoke the experiences of being a female referee or coach including gendered entry into football, women's difference on the football field, and strategies for remaining in the game. By occupying a marginal position in a male-dominated space, this study illuminates the lone challenge for women as the greatest proponents of change in a hegemonically masculinized context.

Given the particular engagement of women in community sport level coaching, Hogan et al. were interested in the influence of the community-based sports club environment on the support and development of volunteer women coaches. Using a creative non-fiction approach, the authors relate the perspectives of three profiles of coaches (novice, experienced, player-coach) in Gaelic football based on interviews with 11 women in Ireland. Vignettes of each type of coach are used to relate the influence of retention and recruitment practices, support structures (communication, training) and club culture and norms (in-groups, traditions, unconscious bias) on their personal development as coaches. A final “letter” from the coaches to the club presents recommendations for building a more inclusive and equitable future for women coaches.

## Author Contributions

All authors listed have made a substantial, direct, and intellectual contribution to the work and approved it for publication.

## Conflict of Interest

The authors declare that the research was conducted in the absence of any commercial or financial relationships that could be construed as a potential conflict of interest.

## Publisher's Note

All claims expressed in this article are solely those of the authors and do not necessarily represent those of their affiliated organizations, or those of the publisher, the editors and the reviewers. Any product that may be evaluated in this article, or claim that may be made by its manufacturer, is not guaranteed or endorsed by the publisher.

## References

[B1] Adams C. Leavitt S. (2018). 'Its just girls hockey: Troubling progress narratives in girls and women's sport. Int. Rev. Sociol. Sport 53, 152–172. 10.1177/1012690216649207

[B2] Casey M. Fowlie J. Charity M. Harvey J. Eime R. (2019). The implications of female sport policy developments for the community-level sport sector: a perspective from Victoria Australia. Int. J. Sport Policy Polit. 11, 657–678. 10.1080/19406940.2019.1618892

[B3] Eime R. Pankowiak A. Casey M. Harvey J. Westerbeek H. (2021). Women and Girls' Participation in Male-Dominated Sports. Available online at: https://www.vu.edu.au/sites/default/files/women-and-girls-participation-in-male-dominated-sports-research-summary.pdf

[B4] Elliott S. Bevan N. Litchfield C. (2020). Parents, girls' and Australian football: a constructivist grounded theory for attracting and retaining participation. Qual. Res. Sport Exerc. Health 12, 392–413. 10.1080/2159676X.2019.1602560

[B5] LaVoi N. M. (2016). A framework to understand experiences of women coaches around the globe. The Ecological-Intersectional Model, in Women in Sports Coaching, 1st ed, LaVoi N. M. (Ed.). London: Routledge. 10.4324/9781315734651-2

[B6] Litchfield C. Elliott S. (2020). Maximising enjoyment to sustain girls' sport participation: a unique case study of netball in Australia. Qual. Res. Sport Exerc. Health 2020, 1–19. 10.1080/2159676X.2020.1778063

